# Note for the Essay, "On the Mortality of Children during the First Dentition," in the Journal of Dental Science, for April, 1853

**Published:** 1854-01

**Authors:** J. L. Levison


### ARTICLE VIII.

*Note for the Essay, "On the Mortality of Children during the First Dentition,"* in the Journal of Dental Science, for April, 1853. By J. L. Levison, D. D. S.

In the paper, published in your valuable Journal on this subject in April, 1853, we traced disturbance during the formative process of the teeth, which especially involved the vascular, the nervous and the mucous systems, and we then promised to add a few remarks on the effects produced on the *dermoid* tissues.

Every practitioner is familiar with teeth-rash, as it is popularly called,* which rises in small vesicular pustules, isolated, or in patches, and is one of nature's modes of modifying the otherwise greater consequences of dental irritation. The pathology of all forms of *dermoid* affections during the primary dentition, will be found to result from two causes, viz. nervous disturbance, and mucous irritation.

That the brain and the skin may be sympathetically affected, is obvious by the fact, that often when cutaneous affections are suppressed, insanity will be manifested; or when the latter sad affliction exists, it is greatly relieved, and not unfrequently cured by modern physicians, by producing, artificially, extensive cuticular pustules.

Skin diseases, therefore, during dentition, may be regarded as an effort of nature to induce counter-irritation. And when it is remembered that the mucous surfaces (or inner skin) are particularly implicated during the progress of the first dentition, and that there exists a great sympathy between the mucous tissues and the cuticle, we have a rationale of the final cause of

* *Tinea mucosa* (milk scall) is often induced during teething. It differs from *tinea annularis*, true ring-worm, as the latter may be the result of uncleanliness, or inoculation; whilst the first may co-exist with dental irritation *per se*, even with the greatest care and with the most rigid cleanliness.

the effort to counteract the tendency to acute diarrhea by extensive skin-affections, which otherwise might terminate fatally. So then it is essential during the first dentition, for the practitioner to mark the various seats of irritation, and to prevent any extreme disturbance of the *vascular* and *nervous* systems, or the *mucous* and *dermoid* tissues, by a free use of the lancet, and, so to speak, go to the source; and by removing the cause of irritation, avoid the many unpleasant and often fatal consequences which result, if these preventive measures are neglected.

